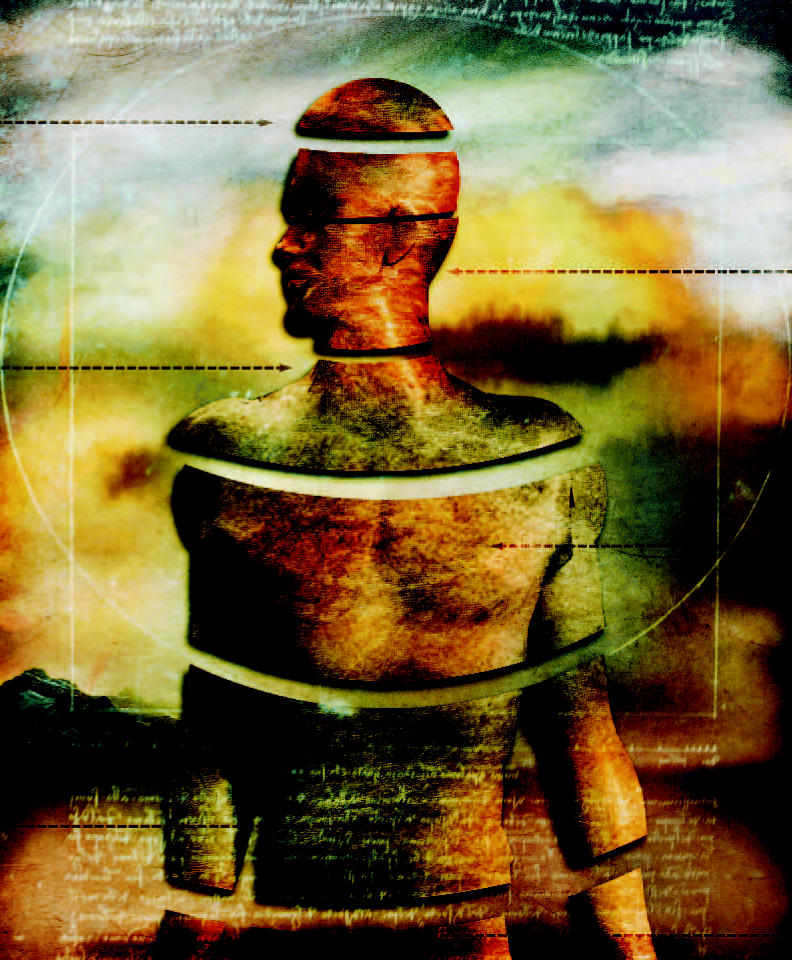# Nanotechnology: Looking As We Leap

**DOI:** 10.1289/ehp.112-a740

**Published:** 2004-09

**Authors:** Ernie Hood

Since 1989, when IBM researchers whimsically demonstrated a scientific breakthrough by constructing a 35-atom depiction of the company’s logo, the ability to manipulate individual atoms has spawned a tidal wave of research and development at the nano (from the Greek word for “dwarf”) scale. Nanomaterials are defined as having at least one dimension of 100 nanometers or less—about the size of your average virus. Nanotechnology—the creation, manipulation, and application of materials at the nanoscale—involves the ability to engineer, control, and exploit the unique chemical, physical, and electrical properties that emerge from the infinitesimally tiny man-made particles.

Nanoparticles behave like neither solids, liquids, nor gases, and exist in the topsy-turvy world of quantum physics, which governs those denizens small enough to have escaped the laws of Newtonian physics. This allows them to perform their almost magical feats of conductivity, reactivity, and optical sensitivity, among others.

“That’s why nanomaterials are useful and interesting and so hot right now,” says Kristen Kulinowski, executive director for education and policy at the Rice University Center for Biological and Environmental Nanotechnology (CBEN). “Being in this quantum regime enables new properties to emerge that are not possible or not exhibited by those same chemicals when they’re much smaller or much larger. These include different colors, electronic properties, magnetic properties, mechanical properties—depending on the particle, any or all of these can be altered at the nanoscale. That’s the power of nanotech.”

Many observers not normally given to hyperbole are calling nanotechnology “the next Industrial Revolution.” The National Nanotechnology Initiative (NNI), the interagency consortium overseeing the federal government’s widespread and well-funded nanotechnology activities, has predicted the field will be worth $1 trillion to the U.S. economy alone by 2015—or sooner. Clearly, nanotechnology is poised to become a major factor in the world’s economy and part of our everyday lives in the near future. The science of the very small is going to be very big, very soon.

## The Springboard

The first swells presaging the approaching nanotechnology tidal wave have already reached the shore. Engineered nanoparticles are already being produced, sold, and used commercially in products such as sporting goods, tires, and stain-resistant clothing. Engineered nanomaterials designed to provide nontoxic, noncorrosive, and nonflammable neutralization of chemical spills or chemical warfare agents are currently on the market. Even sunscreens have gone nano—some now contain nanoscale titanium dioxide or zinc oxide particles that, unlike their larger, opaque white incarnations, are transparent, while still blocking ultraviolet rays effectively. Fullerenes, which are used in commercial products from semiconductors to coatings for bowling balls, are being produced by the ton at a Mitsubishi plant in Japan.

Within a few years, experts say, these initial market forays will seem as quaint as eight-track tapes. According to Mihail Roco, senior advisor on nanotechnology to the National Science Foundation (NSF) and coordinator of the NNI, nanotechnology will have four generations, or phases of development. We’re already in the first, consisting of “passive” nanostructures—simple particles designed to perform one task. Roco predicts the second phase will start in 2005, with the appearance of commercial prototypes of “active” nanostructures such as special actuators, drug delivery devices, and new types of transistors and sensors.

As evidence of progress toward this second phase, a team of Northwestern University chemists led by Chad Mirkin recently announced that they have discovered ways to precisely construct nanoscale building blocks that assemble into flat or curved structures. The ability to create unusual nanostructures such as bundles, sheets, and tubes holds promise for new and powerful drug delivery systems, electronic circuits, catalysts, and light-harvesting materials.

By 2010, Roco says, the third generation will arrive, featuring nanosystems with thousands of interacting components. And a few years after that, the first “molecular” nanodevices will appear, devices that will be composed of systems within systems operating much like a cell.

As manufacturing methods are perfected and scaled up, nanotechnology is expected to soon pervade, and often revolutionize, virtually every sector of industrial activity, from electronics to warfare, from medicine to agriculture, from the energy we use to drive our cars and light our homes to the water we drink and the food we eat. Nanotechnology is today’s version of the space race, and countries around the globe are enthusiastically pouring billions of dollars into support of research, development, and commercialization.

In terms of the environment and human health, nanotechnology presents the same conundrum as past major technological advances: there may be enormous benefits in terms of benign applications, but there are inherent risks as well. What will happen when nanomaterials and nanoparticles get into our soil, water, and air, as they most assuredly will, whether deliberately or accidentally? What will happen when they inevitably get into our bodies, whether through environmental exposures or targeted applications? The answers to those vital questions remain largely unanswered, although some early findings are less than reassuring, as evidenced by a recent study implicating fullerenes in oxidative stress in the brains of large-mouth bass [see “Fullerenes and Fish Brains: Nanomaterials Cause Oxidative Stress,” *EHP* 112:A568 (2004)].

Questions of another sort also need to be answered. Is anyone looking at these health and safety issues? And can enough solid, reliable risk assessment knowledge be gained in time to ensure that the public will—or even that it should—be comfortable with the proliferation of the technology? Will the paradigm shift smoothly into the nano world, or will issues of safety and trust surround nanotechnology with controversy that may hinder its potential, as has happened in the past with such achievements as genetically modified organisms (GMOs)?

Kulinowski expresses the nearly universal sentiments of the field’s advocates: “We think nanotechnology has enormous potential to benefit society in a whole variety of sectors and applications, from the next cancer treatment, to environmental applications, to energy—you name it. So we don’t want to see that potential limited or eliminated by real or perceived risk factors associated with engineered nanomaterials.” To ensure that nanotechnology flourishes responsibly and with strong public support, Kulinowski says, advocates believe it’s very important to gather risk data so that questions can be answered and problems addressed early on in the trajectory of the technology development.

Sean Murdock, executive director of the NanoBusiness Alliance, a nanotechnology trade association, thinks it is possible to avoid past mistakes of rolling out a new technology too far ahead of health and safety information. “The risks are there, they’re real, but they’re manageable,” he says. “And on balance, with the right processes in place, we’re going to be able to deal with all of those risks, we’re going to mitigate those risks, and we’re going to realize the upside of the potential.”

## Nanomedicine: A Tiny Dose for Health

One of the most promising applications of nanotechnology, known as nanomedicine, involves the development of nanoscale tools and machines designed to monitor health, deliver drugs, cure diseases, and repair damaged tissues, all within the molecular factories of living cells and organelles. The NIH Roadmap for Medical Research—the agency’s master plan to accelerate the pace of discovery and speed the application of new knowledge to biomedical prevention strategies, diagnostics, and treatments—contains a significant nanomedicine initiative that will begin with the establishment of 3–4 Nanomedicine Development Centers. These multidisciplinary facilities will serve as the intellectual and technological centerpiece of the endeavor. Funding for the centers of $6 million per year will begin in September 2005.

Today, the initiative’s long-term goals sound like scenarios straight out of Isaac Asimov’s *Fantastic Voyage*: nanobots that can search out and destroy cancer cells before they can form tumors . . . nanomachines that can remove and replace broken parts of cells . . . molecule-sized implanted pumps that can deliver precisely targeted doses of drugs when and where they’re needed . . . even “smart” nanosensors that can detect pathology or perturbation in any or every cell in the body, and instantly communicate that information to doctors. Science fiction may soon become science fact—these and many other nanomedicine innovations are currently in development, and the NIH predicts that its nanomedicine initiative will start yielding medical benefits in as soon as 10 years. Roco also foresees that fully half of all drug discovery and delivery technology will be based on nanotechnology by 2015.

Experts predict that nanosensors will also provide significantly improved tools to determine both internal and external exposures in real time, assess risk, link exposure to disease etiology, characterize gene–environment interactions, and ultimately improve public health. The NIEHS, through its extramural grants and Superfund Basic Research Program, is funding the research and development behind many of these expected innovations.

For example, with a Small Business Innovation Research grant, the institute is supporting Platypus Technologies of Madison, Wisconsin, in its work on smart nanosensors designed to act as personal dosimeters for real-time and cumulative exposure to toxic compounds. Combining scaled-down photo optics and nanomaterials to form a uniquely sensitive platform for exposure detection, the initial prototype device is intended to detect even very low exposures to organophosphate pesticides. The sensor, expected to be available commercially within two years, is small, lightweight, passive, inexpensive, and easily operated—one immediate application will be monitoring the chemical environments of children.

Platypus CEO Barbara Israel elaborates: “Our product is ‘tunable’ for different anticipated concentration ranges and monitoring time periods. Therefore, it can be applied to monitor workers for occupational exposure to toxic compounds during manufacturing, as well as to the monitoring of field exposure of agricultural workers.” The company is also developing sensors that will immediately respond to the ambient presence of very low concentrations of other toxic agents, and expects that units will be networked by the thousands in security systems at facilities such as airports and train stations, as well as having industrial applications.

“This technology is going to revolutionize how we do business”—the business of environmental health science, that is—according to William Suk, director of the Center for Risk and Integrated Sciences within the NIEHS Division of Extramural Research and Training. Suk oversees many of the institute’s extramural grants involving nanotechnology. “One of the real potentials of this technology is to truly be able to understand gene–environment interactions, to be able to take the ‘omics’ revolution and scale it down in such a way that you have a comprehensive global approach to understanding how things fit together,” he says. “We’re really looking at the use of these technologies in systems biology, to understand how systems communicate—how cells communicate amongst themselves, and within themselves, and with other cell systems within our body. It’s all connected.”

A wide variety of extraordinarily sophisticated nanobiosensors fitting Suk’s vision are well along in development among institute grantees. For example, neurotoxicologist Martin Philbert at the University of Michigan is perfecting a sensor that measures and identifies chemical perturbations within the mitochondria of neurons, and may eventually allow intervention or prevention of such cellular disturbances. Roger Tsien, a professor of pharmacology, biochemistry, and chemistry at the University of California, San Diego, is developing toxicity sensors that can indicate exposures and the perturbations they cause at the genomic level in real time. Kenneth Turtle-taub, a scientist at Lawrence Livermore National Laboratory, uses an accelerator mass spectrometer to look at nanostructures for biomarkers of exposure to carcinogenic chemicals, characterizing perturbations at the atomic level. According to Suk, these and other nanodevices will be making major contributions to the field of environmental health within the next five years. When nanotechnology achieves its full impact, he says, toxicogenomics will evolve beyond its infancy and begin to fulfill its promise of significant improvements in public health.

## Small Improvements in A Big World

Although research and development of environmental applications is still a relatively narrow area of nanotechnology work, it is growing rapidly, and nanomaterials promise just as dazzling an array of benefits here as they do in other fields. Nanotechnology will be applied to both ends of the environmental spectrum, to clean up existing pollution and to decrease or prevent its generation. It is also expected to contribute to significant leaps forward in the near future in environmental monitoring and environmental health science.

The Science To Achieve Results (STAR) program of the U.S. Environmental Protection Agency (EPA), administered by the agency’s National Center for Environmental Research, was an early investor in and promoter of environmental applications of nanotechnology. Beginning in 2001, the agency devoted a small discretionary portion of its grant-making budget to nanotechnology. “We decided to do applications with respect to the environment first,” says Barbara Karn, who oversees the nanotechnology aspect of the program. “We wanted to make a case for the new technology being useful for the legacy issues of EPA.”

Contaminated soil and groundwater are among the most prominent of those legacy issues, and there has been considerable progress in nanotechnology-based remediation methods. Environmental engineer Weixian Zhang of Lehigh University, a STAR grantee who also receives funding from the NSF, has been working since 1996 to develop a remediation method using nanoscale metallic particles, particularly iron nanoparticles, which he has found to be powerful reductants. “If any contaminant can be degraded or transformed by reduction,” he says, “you can use the iron nanoparticles.” He has been field-testing the method since 2000, both in pilot studies and at several industrial sites contaminated with such toxicants as polychlorinated biphenyls, DDT, and dioxin, and the results have been encouraging.

Zhang’s nanoremediation offers several potential advantages over existing methods. The implementation is very simple—the nanoparticles are suspended in a slurry and basically pumped directly into the heart of a contaminated site. By comparison, current methods often involve digging up the soil and treating it.“You can inject [the nanoparticles] in some difficult situations, for example, under a runway, under a building, or other sites where typical engineering methods may not be feasible,” says Zhang.

Nanomaterials have a large proportion of surface atoms, and the surface of any material is where reactions happen. Because of nanoparticles’ huge surface area and thus very high surface activity, workers can potentially use much less material. The amount of surface area also allows a fast reaction with less time for intermediates to form—a boon in biodegradation, where the intermediate products are sometimes more toxic than the parent compound. Finally, Zhang’s method is also much faster. “Because of the higher activity, it takes much less time to achieve remediation goals than conventional technology, which, using biological processes, can take years,” he says. With the iron nanoparticles, in most cases the team saw contaminants neutralized into benign compounds in a few days.

Zhang is currently focusing on scaling up production of the iron nanoparticles to make them more cost-competitive, and plans to establish a business based upon his techniques. His is just one of dozens of nanoremediation methods being developed, but is probably the closest to large-scale deployment—he expects that within a year or two, there will be tens to hundreds of projects using the metallic nanoparticle technology. And this type of “passive” application is only the beginning.

“In the future,” says Zhang, “we’ll have more sophisticated devices that can function not only as a treatment device, but also as a sensor with detection functions and communication capability that you can put into the ground and get feedback on different environmental parameters.” That type of device will give remediators the ability to determine when a treatment has been adequately completed, currently a problematic determination to make. Similar nanosensors that will allow real-time *in situ* detection and analysis of pollutants are being developed for environmental monitoring purposes.

The environmental benefits portended by nanotechnology go farther still. Improvements in membrane technology afforded by nanomaterials, for example, will allow greatly enhanced water filtration, desalination, and treatment of wastewater through finer and “smarter” selective filtration. The technology that is expected to be proliferated is also anticipated to be very simple and very inexpensive. These developments are expected to eventually go a long way toward ameliorating the shortages of clean, plentiful, low-cost drinking water that plague many areas of the world.

Murdock says nanotechnology is also likely to help prevent a great deal of pollution in the future by affording the opportunity to “reinvent the energy infrastructure that powers the economy, which ultimately has been driving many of the issues that environmentalists . . . have been worrying about over the past few decades.” Nanoscale materials and devices could result in game-changing breakthroughs in energy production through advances in hydrogen and solar energy, and could even beget vast improvements in the efficiency and cleanliness of carbon-based energy. There is serious talk, for example, that nanotechnology could make it possible to sustainably expand the use of coal in energy production, using a nanocatalyst that turns coal directly into cleaner-burning diesel fuel and gasoline.

On the other hand, nano-based lighting is already a reality—traffic lights across the country now use tiny light-emitting diode displays that remain in service longer and use less energy than bulbs. The NNI has projected that widespread proliferation of the technology for home and office lighting could cut U.S. energy consumption by as much as 10%, dropping carbon emissions by up to 200 million tons annually.

With their extremely high reactivity, nanomaterials may also enable “green” chemistry and “exact” manufacturing, in which chemicals and other products are manufactured from the bottom up, atom by atom. This development would allow the creation of less-toxic products while reducing or eliminating both hazardous waste and the need for large quantities of toxic raw materials—so-called source reduction. The green chemistry concept applies to the production of nanoparticles themselves. University of Oregon chemist James Hutchinson recently patented a more benign (and faster and cheaper) method for producing gold nanoparticles, which are particularly important in the semiconductor industry.

Karn is excited by this and similar developments: “We really have such an opportunity here with this new technology, to make it without waste, to make the particles in an environmentally friendly way, so that we don’t have to worry about the emissions [and] we don’t have to worry about the cleanup afterwards.”

## A Yellow Light

The same properties that confer such incredible utility to engineered nanoparticles are those that raise concerns about the nature of their interactions with biological systems: their size, their shapes, their high reactivity, how they are coated, and other unique characteristics could prove to be harmful in some physiologic circumstances. Several recent studies have appeared in the literature showing that some nanomaterials are not inherently benign. Some can travel readily through the body, deposit in organ systems, and penetrate individual cells, and could trigger inflammatory responses similar to those seen with ambient nanoparticles—better known in environmental science as ultrafine particles—which are known to often be far more toxic than their larger counterparts. The primary difference between ambient and engineered nanoparticles is that the former have widely varying shapes, sizes, and compositions, whereas the latter are single, uniform compounds.

University of Rochester environmental toxicologist Günter Oberdörster has shown in rodent studies, published in June 2004 in *Inhalation Toxicology*, that inhaled nanoparticles accumulate in the nasal passages, lungs, and brains of rats. And in the January 2004 issue of *Toxicological Sciences*, National Aeronautics and Space Administration scientist Chiu-Wing Lam recently reported that a suspension of carbon nanotubes (one of the most widely used and researched engineered nanoparticles) placed directly into mouse lungs caused granulomas, unusual lesions that can interfere with oxygen absorption. David Warheit, a DuPont researcher, conducted a similar experiment in rats, reported in the same issue of *Toxicological Sciences*, and discovered immune cells gathering around clumps of nanotubes in the animals’ lungs. At the highest dose, 15% of the rats essentially suffocated due to the clumping of the nanotubes having blocked bronchial passages. Although Lam’s and Warheit’s studies did not reflect potential real-world exposures, their results were nonetheless troubling, showing at least that nanotubes are biologically active and possibly toxic.

A study published in the July 2004 issue of *EHP* documenting oxidative stress (a sign of inflammation) in the brains of largemouth bass exposed to aqueous fullerenes has received perhaps the most attention and raised the most warnings of any nanomaterial health implication experiment to date. Eva Oberdörster (Günter’s daughter), an environmental toxicologist at Southern Methodist University, describes herself as “shocked” at the amount of mainstream national press coverage the study has received. She is quick to stress that although some reports have described “brain damage” or even “severe brain damage” in the fish, she has actually characterized her findings as “significant damage in the brain, which is very different from brain damage.” After 48 hours’ exposure to fairly high doses of fullerenes, the fish probably had the same effect as a very bad headache, she says, but they did survive the exposure. As to the inflammation, Oberdörster says it could have been an appropriate response to a foreign stressor or a symptom of real physiologic damage. She plans to study this issue further in gene microarray experiments designed to more thoroughly characterize the inflammatory response involved, and to see whether the fish might actually metabolize and excrete the particles.

Oberdörster has described the findings as “a yellow light, not a red one,” and explains further that there are some indications from the inhalation and fish studies that there is a potential for nanoparticles to react with tissues and create inflammation. “So the next step then is to look at it in a broader spectrum before we bring all these products out into the market, to make sure that they are safe so that consumers are protected,” she says.

Kulinowski feels that the early studies raise more questions than answers, and she cautions against overinterpreting individual studies. She is optimistic, however, that with technological advances, the potential negative impacts of engineered nanoparticles can be minimized or eliminated altogether. “The good news I see is that with the control we have over engineered nanoparticles, we may be able to engineer them to confer the benefits, but not the risks, not the hazards.” Again, it’s all about the surface, she says: “If we can control surface properties of nanoparticles, we may be able to tune out the toxicity. . . . It’s like sliding a dimmer switch on a lamp—you can just tune it right down to pretty much beyond our capacity to measure it.”

## Big Issues for Nanotechnology

Nanotechnology accomplishments could soon affect every person on the planet. But the opinion is virtually unanimous, among advocates and skeptics alike, that the full realization of nanotechnology’s potential benefits is threatened by ongoing concerns about the potentially negative effects nanomaterials could have on human health and the environment. Applications are being vigorously pursued. The question is, will knowledge of the implications keep pace?

In August 2002, the Action Group on Erosion, Technology, and Concentration (ETC Group), a Canadian environmental activist group that played a key role in the battle against acceptance of GMOs in the 1990s, called for a worldwide moratorium on research and commercialization of engineered nanomaterials until there are protocols in place to protect workers, including lab workers. They cited a dearth of research data about the potential negative implications of nanomaterials, and the lack of specific regulatory oversight or established best practices in the handling of nanoparticles in either the laboratory or the manufacturing setting.

Perhaps coincidentally, in the two years since the ETC Group’s moratorium call there has been a palpable upsurge in research and bureaucratic activity regarding those missing pieces of the puzzle. All of the stakeholders, casting a weather eye at the GMO experience, apparently agree that the bountiful benefits of nanotechnology cannot be harvested without full and transparent characterization of the risks they could pose to human health and the environment.

“We’re much better off over the long haul if we make sure that we address concerns and issues proactively,” says Murdock. “That doesn’t mean we should be hypersensitive and shy away from exploring new areas, because fundamentally we only make progress through exploration. But it needs to be balanced and tempered with a continuous examination of the implications.”

Roco, who has been instrumental in the NNI’s ongoing attention to both safety implications and the potential societal impacts of nanotechnology worldwide, agrees that the time for responsible risk assessment is now: “This is no longer something you do after the fact, after you do the other research, but has to be done from the beginning, to be an integral part of the research. You have to look at the whole cycle of activity, not only at the first phase when you create something.”

The CBEN has been investigating the environmental fate of nanoparticles since its inception in 2001 as one of six Nanoscale Science and Engineering Centers established by the NSF, and Kulinowski has noted the recent explosion in interest and funding in nanotech environmental health and safety research. “We have seen tremendous movement on this issue over the last year and a half, from the point where we almost felt like we were calling out into the darkness, to where people are now moving forward independently,” she says. “Most encouraging has been the federal government’s response. We’ve also seen a tremendous response from industry . . . that gives us hope that as we move toward commercialization of nanotechnology products, these questions will be addressed early on in the development, before or when products come to market.”

Critics and even some participants maintain that funding of implications research is still inadequate in proportion to the nearly $1 billion the government is currently investing in nanotechnology development. But efforts to better understand the implications of nanotechnology are clearly gaining momentum. Important new research initiatives are getting under way, and coordination and collaboration are increasing among the federal regulatory agencies and research organizations involved.

The NNI is the central locus, with a number of agencies participating. Representatives from several of those agencies, including the NIEHS, the EPA, and the National Institute for Occupational Safety and Health (NIOSH), have formed a working group on the environmental and health impacts of nanotechnology. The group meets monthly to share knowledge, coordinate activities, identify research gaps and goals, and address urgent issues such as regulation and nomenclature.

Two further major research initiatives designed to establish fundamental knowledge about the toxicologic properties of engineered nanomaterials are in their initial stages. Both will contribute significantly to the knowledge base and allow more rational risk assessment in the future.

The first of these major initiatives arose from the CBEN’s 2003 nomination of nanomaterials for study by the National Toxicology Program (NTP). The NTP, which is headquartered at the NIEHS, has embarked upon a research program involving safety studies of representative manufactured nanomaterials. “The aim of our program,” says Nigel Walker, lead scientist of the investigation, “is actually to help guide the nanomaterial industry in identifying the key parameters that lead to biocompatibility of nanomaterials, versus toxicity of nanomaterials, so that we can avoid having problems such as the genetically modified food situation, where the industry and the technology got ahead of the biocompatibility issues.”

The NTP program will focus initially on studies of single-walled carbon nanotubes, titanium dioxide, quantum dots (fluorescent semiconductor nanocrystals used in imaging equipment), and fullerenes. Because the most likely route of exposure to those nanomaterials as they are used today is through the skin, several studies will concentrate on dermal toxicity. Other exposure routes will be examined as well, however, all looking at general, acute, subchronic, and chronic levels of exposure.

One of the broad goals of the NTP initiative is to create models of nanomaterial chemical, physical, and pharmacokinetic properties that can be used to help evaluate new engineered nanostructures as they come along. According to John Bucher, deputy director of the NIEHS Environmental Toxicology Program, the purpose of this initiative is not to prevent or understand the toxicity of every material that can be manufactured under the “nano” rubric. Instead, he says, “What we’re trying to do is understand some of the fundamental properties of nanomaterials—how they move, what kind of toxicities they have, what kinds of organ systems are generally targeted, what the effects of surface coatings are. . . . We’re not trying to make the world safe from nanotechnology, nor do we believe that the world is necessarily at great risk from nanomaterials at this moment, or potentially even in the future. But the total absence of any information makes this an area that we just have to pursue.”

The NTP, in association with the University of Florida, is also planning a workshop for November 2004 designed to bring together scientists from the toxicology community, environmental engineers, and representatives of the pharmaceutical and chemical industries. The workshop will focus on questions about how best to assess exposure to nanomaterials and evaluate their toxicity and safety.

Walker thinks these efforts are timed perfectly. “If we’d tried to do this two or three years ago, we may actually have been targeting things that weren’t important,” he says. “You don’t want to be too early on the curve, but then you don’t want to be too late. This is about the right time . . . and we are being very open about how things are moving along, because the NTP is completely open, and all the data is ultimately the public’s.”

The second major initiative—research on occupational health risks associated with manufacturing and using nanomaterials—is being spearheaded by NIOSH. The institute recently organized a Nanotechnology Research Center to coordinate, track, and measure outcomes, and disseminate the output of nanotechnology-related activities throughout the institute.

NIOSH has also undertaken a five-year multidisciplinary initiative known as the NIOSH Nanotechnology and Health and Safety Research Program. As with the NTP’s efforts, the idea is to characterize risks early in the industry’s development, and the workplace is the most likely location of exposures at present. “There is some concern that these materials are of unknown effect, and there is interest in getting generalized industrial hygiene, generalized control measures, and best work practices involved early on,” says researcher Vincent Castranova, who is principal investigator for the program’s coordinating project as well as a separate study exploring particle surface area as a dose metric. “Normally, interest in these elements has come after proof of disease outcome. This is one case where the concerns are sufficient to cause the industry and the governmental agencies to try to get good work practices and prevention measures up front, before we know full health outcomes.”

Another NIOSH scientist, Andrew Maynard, is investigating methods of characterizing and monitoring airborne nanoparticles. “Part of my project,” says Maynard, “is developing and using the characterization techniques, so that we can understand very precisely the chemical and physical nature of the particles, and also the concentration of the particles being used in these experiments. Also, we will look at how we can effectively monitor exposures in the workplace, so that we can have simple, robust, inexpensive techniques that people can use in the workplace.”

Although dermal exposure to nanomaterials is occurring as a result of their use in sunscreens and some cosmetics, inhalation is suspected to be the most likely route of exposure in the workplace, so other projects in the program will focus on pulmonary toxicity questions, particularly with respect to carbon nanotubes. Those questions will be tricky, again due to the unique attributes of nanomaterials—they are technically ultrafine particles, but can they be judged the same way?

“This is one of the big areas of debate at the moment,” says Maynard. “To what extent do you treat engineered nanoparticles as just another ultrafine particle? It’s fair to say that most of our concerns over nanomaterials are being driven by our experiences with ultrafine particles, which are substantially more inflammatory and toxic than fine particles.”

“Another issue that is unresolved is that these nanoparticles tend to aggregate, and the aggregates often tend not to be under a hundred nanometers in diameter,” adds Castranova. “So do they then behave as fine particles rather than ultrafine? That depends on whether they disaggregate either in handling or once they’re in the lung, which is unknown. Their ability to enter the lungs, to cross the air–blood barrier, or to cause inflammation would be affected by [disaggregating].” NIOSH is helping to organize the First International Symposium on Occupational Health Implications of Nanomaterials, which will convene in the United Kingdom in October to discuss these issues.

## The Ripple Effect

The NTP and NIOSH initiatives are the major new programs in the works, but a great deal of activity is continuing or beginning in other circles as well. The NNI is expanding its support of implications research, and recently held a landmark international meeting that brought together the leaders of nanotechnology programs in 25 countries and the European Union. The International Dialog on Responsible Research and Development of Nanotechnology took place 17–18 June 2004 in Arlington, Virginia, and was designed to help develop a global vision of how the technology can be fostered with the appropriate attention to and respect for concerns about the societal issues and environmental, health, and safety implications.

Roco, who called the meeting “a historic event,” proposed the establishment of an ongoing international organization dedicated to responsible nanotechnology development. Participants agreed to form a “preparatory group” charged with exploring possible actions, mechanisms, timing, institutional frameworks, and principles involved in constructing a permanent institution designed to ensure international dialogue, cooperation, and coordination in nanotechnology research and development.

ETC Group executive director Pat Mooney reacted favorably to the gathering as well: “That’s the first time we’ve had an international meeting like this, and I think it’s a very encouraging sign.”

Encouraging signs of commitment and progress were also evident at two other landmark events held earlier this year. In March, the NIEHS held a workshop called Technologies for Improved Risk Stratification and Disease Prevention that brought together a panel of experts to formulate specific recommendations on how the institute should incorporate nanotechnology into its research agenda in the coming years. Participants embraced the idea that the NIEHS should lead the way in developing a single small-scale platform to detect individual chemical exposures, eliminate toxicants from the system, and intervene to reverse any harmful effects that might have been initiated by the exposure. Then, in May, a one-day public discussion was held by the Institute of Medicine’s Roundtable on Environmental Health Sciences, Research, and Medicine, in which experts and members of the public explored the issues raised by nanotechnology from a public health perspective. The discussion illuminated potential public health benefits while acknowledging recent toxicological concerns. Events such as these serve to inform the scientific community and the public alike, encouraging the responsible development of the technology.

Recognizing the enormous opportunities at hand, the chemical industry is also placing a high priority on nanotechnology implications research. A consortium called the Chemical Industry Vision2020 Technology Partnership, in cooperation with the NNI and the U.S. Department of Energy Office of Energy Efficiency and Renewable Energy, released a comprehensive white paper in 2003 titled “Chemical Industry R&D Roadmap for Nanomaterials by Design: From Fundamentals to Function.” This document calls for an unprecedented level of cooperation and collaboration among U.S. chemical companies to foster the long-term success of the nanochemical industry, and stresses that environment, safety, and health knowledge will be an essential component. “The anticipated growth in nanoparticle utilization warrants parallel efforts in hazard identification, exposure evaluation, and risk assessment,” the paper states. “Chemical companies are prepared to serve a major role in this process as leaders in characterizing materials, identifying their potential risks, and providing guidelines for their safe and effective utilization.”

The EPA’s STAR program is planning to award new grants soon in nanotechnology implications research, and the CBEN is continuing its work on what it calls “the wet–dry interface”—the interactions between engineered nanomaterials and systems that are active in aqueous or water-based environments, including ecosystems and living beings. “We have several research projects we would characterize as implications research,” says Kulinowski, “looking at what happens when nanomaterials get into the soil or into a water supply.” By understanding how nanoparticles (which are typically not soluble in water, hence the “dry” side) interact with aqueous environments (the “wet” side), the researchers hope to create technologies that will improve human health and the environment, such as biocompatible nanoparticles or nanostructured catalysts that will break down organic pollutants. The wet–dry interface also plays a major role in determining the environmental fate and transport of nanomaterials.

Regulatory agencies such as the EPA, the FDA, and the Occupational Safety and Health Administration are all participating in the NNI, following the progress of the research carefully, and building their own knowledge bases with an eye toward the eventual development and implementation of nano-specific regulatory frameworks within their purviews. At present the consensus seems to be that existing regulations are sufficiently robust to appropriately address concerns related to nanomaterials, but as risks and hazards are characterized in more detail, that stance could change.

Even the ETC Group, although it has not rescinded its call for a moratorium, seems encouraged by recent progress. “We do feel like we have had a reasonable response—as reasonable as to be expected—from the governments,” says Mooney, “and that there is work under way to try to correct the problems that we have identified.” Mooney says that as individual nations put nano-specific laboratory protocols into place, his group will no longer call for a moratorium in those countries.

It appears that all of this research activity is reaching critical mass at just the right time. The nanotechnology bullet train has left the station with the power to take us to some magical places we’ve barely even dreamed of. Although public distrust of the technology could potentially derail the train, many passengers are hoping that increased understanding of both its potential benefits and dangers will keep it on track and allow the journey toward discovery to continue.

## Nanotech-Knowledge-y

### Centers and Initiatives

#### National Nanotechnology Initiative (NNI)

Consortium of 19 agencies oversees the federal government’s widespread and well-funded nanotechnology activities.

**http://www.nano.gov/**

#### Center for Biological and Environmental Nanotechnology (CBEN)

The CBEN, housed at Rice University in Houston, was begun in 2001 as one of six Nanoscale Science and Engineering Centers established by the NSF.

**http://www.ruf.rice.edu/~cben/**

#### National Institute for Occupational Safety and Health (NIOSH)

NIOSH is particularly interested in nanomaterials with regards to occupational safety and health. This page includes information on the NIOSH Nanotechnology Health and Safety Research Program and the NIOSH Nano technology Research Center.

**http://www.cdc.gov/niosh/topics/nanotech/**

#### NIH Roadmap for Medical Research: Nanomedicine

The NIH Roadmap for Medical Research provides a framework for NIH research priorities in upcoming years. The roadmap contains a significant nanomedicine initiative that includes the establishment of multidisciplinary Nano medicine Development Centers.

**http://nihroadmap.nih.gov/nanomedicine/index.asp**

#### Chemical Industry Vision2020 Technology Partnership

This industry-led consortium aims to accelerate innovation and technology development in the chemical industry. The consortium, in cooperation with the NNI and the US Department of Energy Office of Energy Efficiency and Renewable Energy, released a comprehensive white paper in 2003 called the Chemical Industry R&D Roadmap for Nanomaterials By Design: From Fundamentals to Function.

**http://www.chemicalvision2020.org/pdfs/nano_roadmap.pdf**

#### National Toxicology Program (NTP) Nanotechnology Safety Assessment

The CBEN nominated nanoscale materials for study by the NTP. Based on the nomination, the NTP is developing materials and protocols to test a broad spectrum of nanoscale materials for toxicity in animal models over the next several years.

**http://www.niehs.nih.gov/oc/factsheets/nano.htm**

### Other Resources

#### NanoBusiness Alliance

This nanotechnology trade association is developing a range of initiatives to support and strengthen the nanotechnology business community.

**http://www.nanobusiness.org/**

#### Action Group on Erosion, Technology, and Concentration (ETC Group)

This Canadian group of environmental activists has called for a worldwide moratorium on research and commercialization of engineered nanomaterials. **http://www.etcgroup.org/**

### Recent and Upcoming Events

#### Technology and Environmental Health: Implication of Nanotechnology Public Discussion

Participants at this May 2004 meeting of the Institute of Medicine Roundtable on Environmental Health Sciences, Research, and Medicine discussed human and environmental health implications of nanotechnology as well as legislative and societal issues.

**http://www.iom.edu/subpage.asp?id=19612**

#### The International Dialog on Responsible Research and Development of Nanotechnology

The NNI brought together the leaders of nanotechnology programs from countries around the world at this June 2004 workshop.

**http://www.nsf.gov/home/crssprgm/nano/dialog.htm**

#### First International Symposium on Occupational Health Implications of Nanomaterials

This workshop, convened by NIOSH, the U.K. Health and Safety Laboratory, and the U.K. Health and Safety Executive, will convene in the United Kingdom in October 2004 to discuss workplace issues related to nanomaterials.

**http://www.hsl.gov.uk/news/nanosymp.htm**

The NTP, in association with the University of Florida, is also planning a workshop for November 2004 to focus on questions about how best to assess exposure to nanomaterials and evaluate their toxicity and safety.

## Figures and Tables

**Figure:**
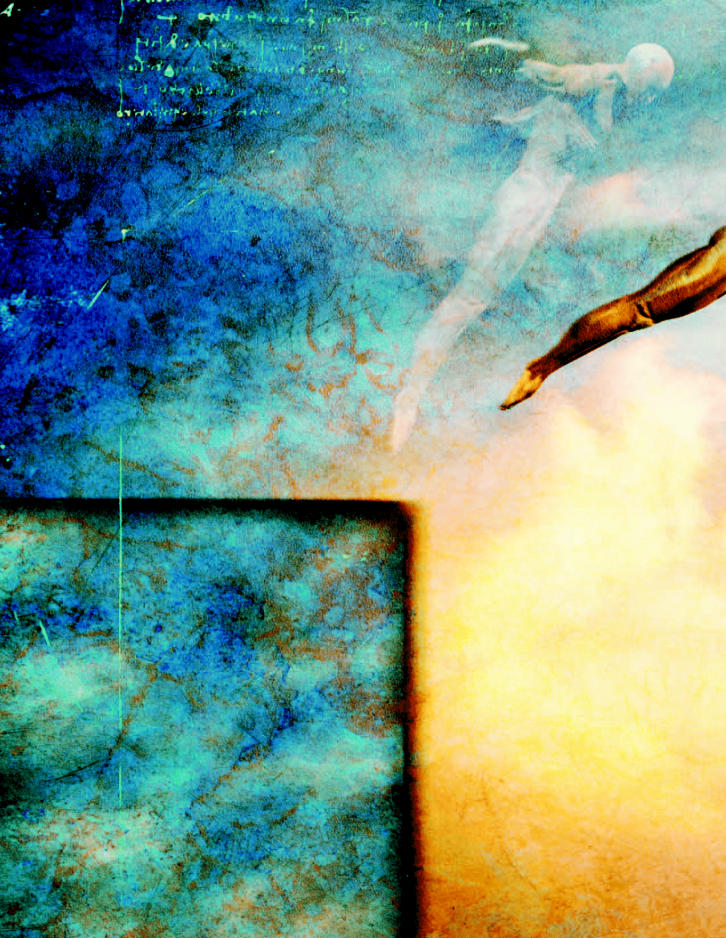


**Figure:**
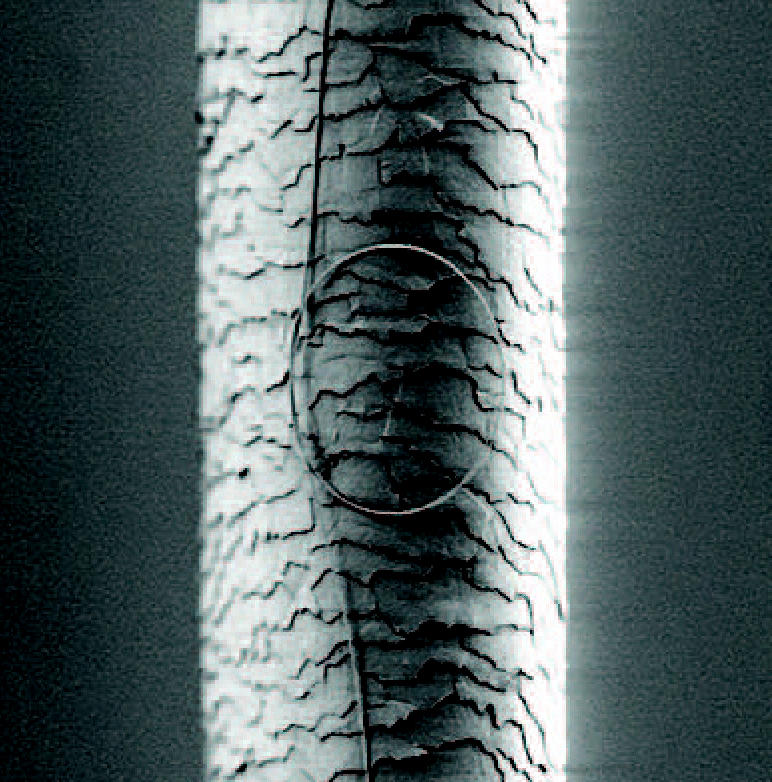
**For comparison’s sake.** A micrograph shows a nanowire curled into a loop in front of a human hair. Nanowires can be as slender as 50 nanometers, about one-thousandth the width of a hair.

**Figure:**
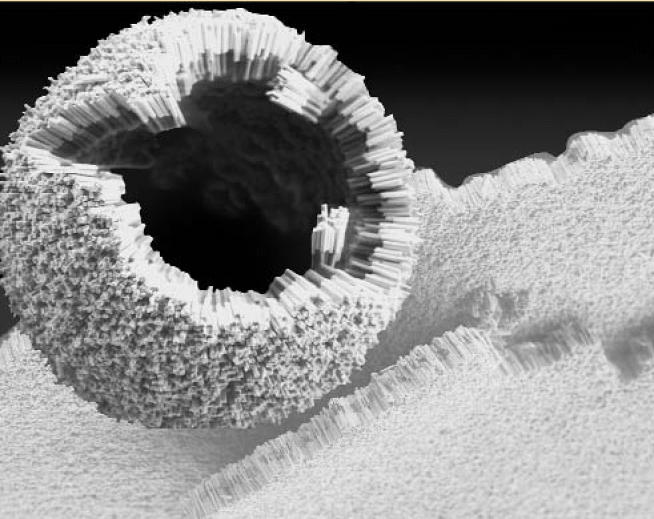
**Small learning curve.** Self-assembly of gold polymer nanorods results in a curved structure. The ability to control the size and curvature of nanostructures could aid in applications in drug delivery and electronics.

**Figure:**
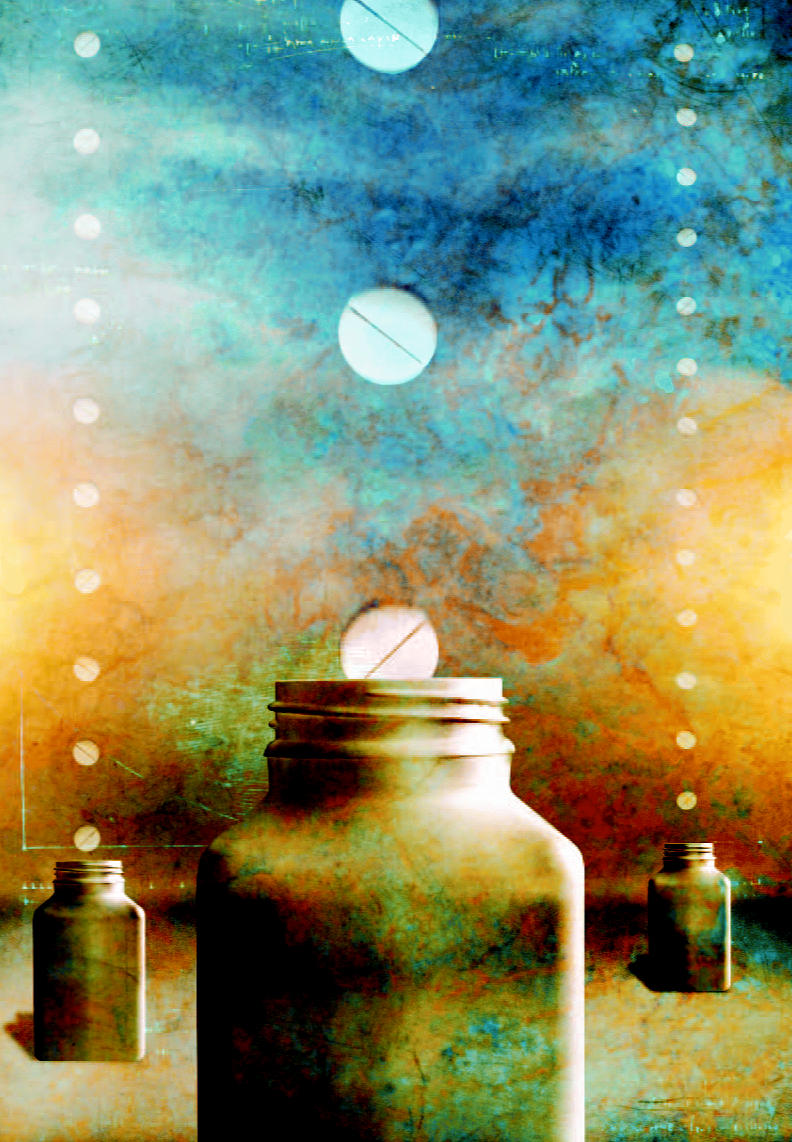


**Figure:**
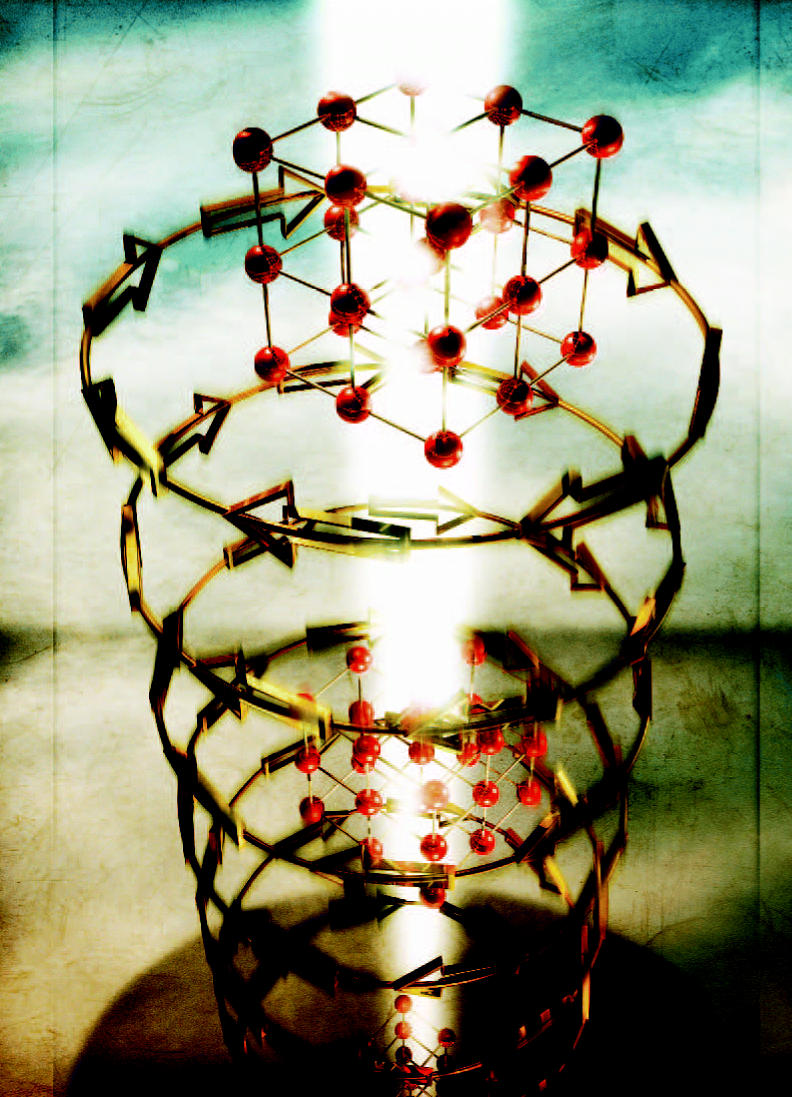


**Figure:**
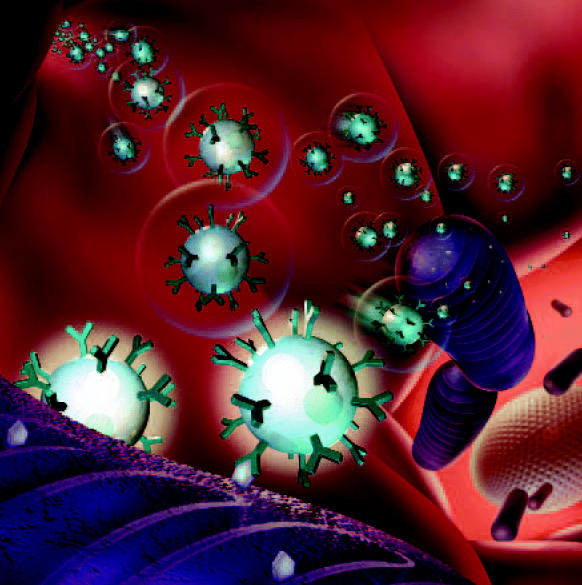
**Probing insights.** Nanoprobes studded with molecules that bind ions such as zinc, calcium, and potassium are injected into cells to reveal the patterns of ion exchange that make cells function. Computer models are used to interpret the fluorescent signatures probes emit when they capture a target ion.

**Figure:**
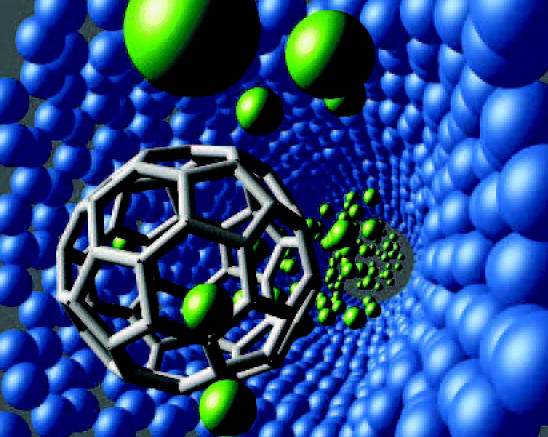
**Shapes and sizes.** A visualization of a nanohydraulic piston consists of common nanotechnology components including a carbon nanotube (blue), helium atoms (green), and a "buckyball" molecule (gray).

**Figure:**
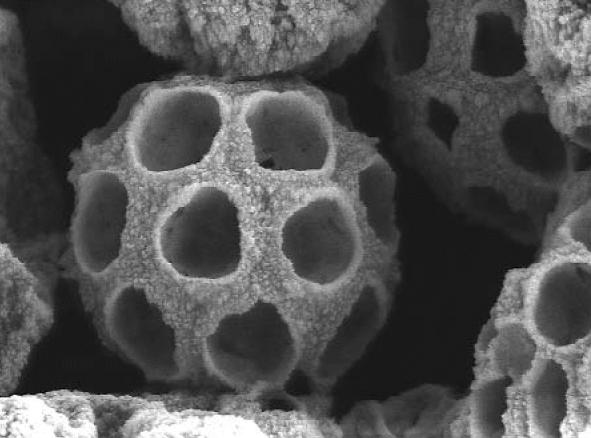
**Tiny beach umbrellas.** A titanium dioxide microsphere (approximately 1–50 microns in diameter) with closed-packed spherical inclusions functions in sunscreens as a small “photonic crystallite,” scattering light very effectively.

**Figure:**